# Maintaining order: COG complex controls Golgi trafficking, processing, and sorting

**DOI:** 10.1002/1873-3468.13570

**Published:** 2019-08-16

**Authors:** Jessica B. Blackburn, Zinia D'Souza, Vladimir V. Lupashin

**Affiliations:** ^1^ Department of Physiology and Biophysics University of Arkansas for Medical Sciences Little Rock AR USA; ^2^ Present address: Division of Allergy, Pulmonary and Critical Care Medicine Department of Medicine Vanderbilt University Medical Center Nashville TN USA

**Keywords:** COG complex, glycosylation, Golgi, SNARE, tethers, vesicular trafficking

## Abstract

The conserved oligomeric Golgi (COG) complex, a multisubunit tethering complex of the CATCHR (complexes associated with tethering containing helical rods) family, controls membrane trafficking and ensures Golgi homeostasis by orchestrating retrograde vesicle targeting within the Golgi. In humans, COG defects lead to severe multisystemic diseases known as COG‐congenital disorders of glycosylation (COG‐CDG). The COG complex both physically and functionally interacts with all classes of molecules maintaining intra‐Golgi trafficking, namely SNAREs, SNARE‐interacting proteins, Rabs, coiled‐coil tethers, and vesicular coats. Here, we review our current knowledge of COG‐related trafficking and glycosylation defects in humans and model organisms, and analyze possible scenarios for the molecular mechanism of the COG orchestrated vesicle targeting.

## Abbreviations

**ARF**, ADP ribosylation factor

**CASP**, CDP/cut alternatively spliced cDNA

**CATCHR**, complexes associated with tethering containing helical rods

**CCD**, COG complex dependent

**CCT**, coiled‐coil tethers

**CDG**, congenital disorders of glycosylation

**CHO**, Chinese hamster ovary

**COG**, conserved oligomeric golgi

**COPI/COPII**, coat protein complex I/complex II

**DKO**, double knockout

**EARP**, endosome‐associated recycling protein

**EELS**, enlarged endolysosomal structure

**EM**, electron microscopy

**ER**, endoplasmic reticulum

**FRAP,** fluorescence recovery after photobleaching

**GARP**, Golgi‐associated retrograde protein

**GEARs**, COG‐sensitive, integral membrane Golgi proteins

**GSL**, glycosphingolipids

**HEK**, human embryonic kidney

**HPA**,* Helix pomatia* agglutinin

**IP**, immunoprecipitation

**KD**, knockdown

**KO**, knockout

**MS,** mass spectrometry

**MTC**, multisubunit tethering complexes

**PI4P**, phosphatidylinositol 4‐phosphate

**PM**, plasma membrane

**SM**, Sly1/Munc18

**SNAP**, soluble NSF attachment protein

**SNARE**, soluble NSF (N‐ethylmaleimide sensitive factor) attachment proteins (SNAP) receptor

**SubAB,** subtilase cytotoxin

**TGN**,* trans*‐Golgi Network

**TM**, transmembrane

**Y2H**, yeast two hybrid

## Intracellular membrane trafficking pathways and machinery

**Figure 1:**
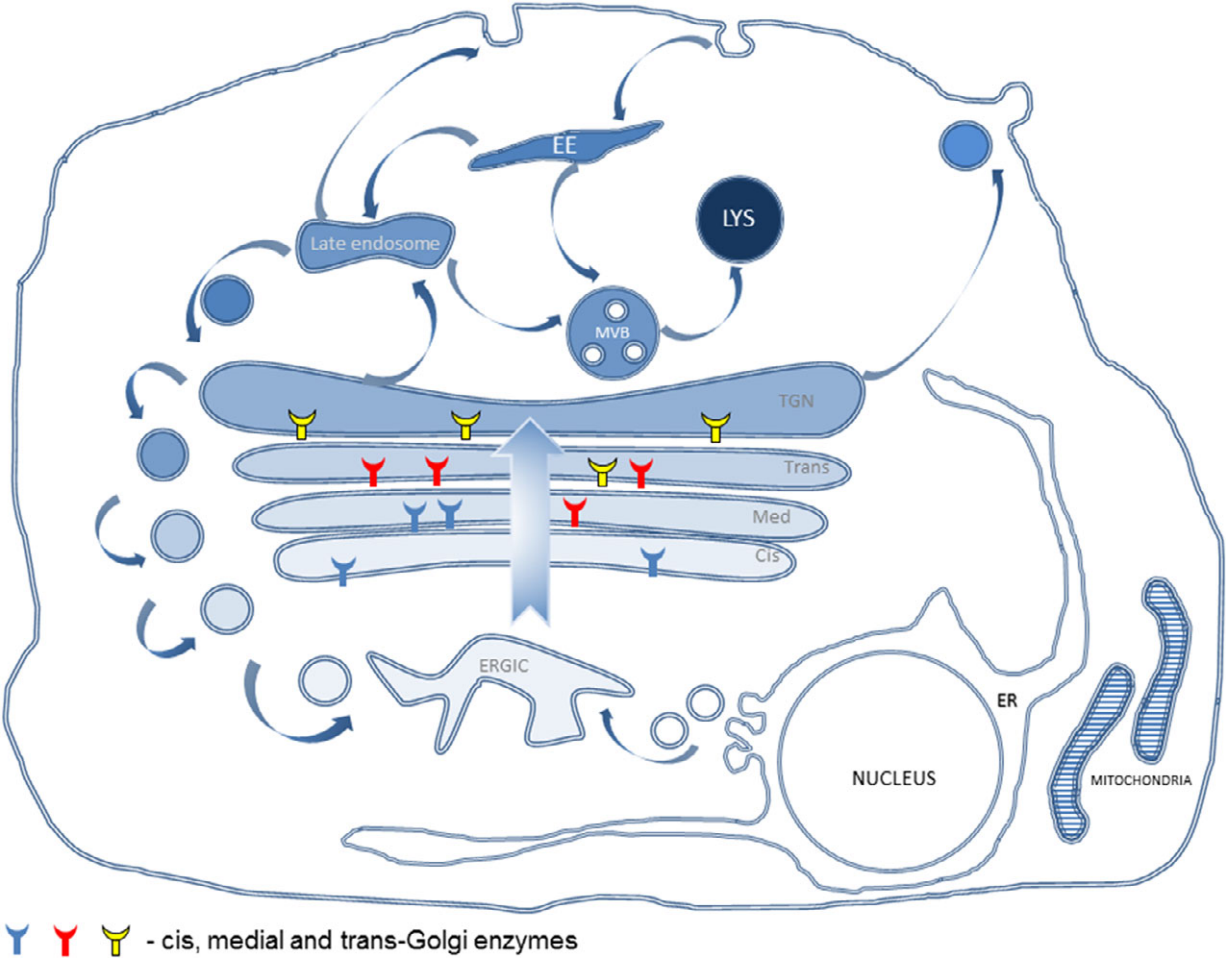
Anterograde and retrograde trafficking pathways and organelles in eukaryotic cell.

Membrane trafficking transports ~ 30% of all proteins through the secretory pathway, a process that governs proper localization of both soluble and membrane‐bound proteins as well as lipids in eukaryotic cells (Fig. 1). Trafficking and post‐translational modifications begin in the endoplasmic reticulum (ER) and continues in the Golgi before cargo is sorted and sent to its final destination. This process is also called anterograde trafficking. Proteins and enzymes that are part of the trafficking and processing machinery also get packaged into transport intermediates during anterograde trafficking. However, it is important that they remain properly compartmentalized, and thus must be returned to their proper location. This is achieved by retrograde vesicular trafficking.

The Golgi makes up < 10% of total cellular membranes but is a central hub for membrane trafficking, receiving a constant flux of membranes from the plasma membrane (PM), endosomes, and the ER. Active anterograde and retrograde trafficking occurs both within and to/from the Golgi, making it a highly dynamic organelle. This balance of anterograde and retrograde trafficking at the Golgi is important for preserving the Golgi structure and maintaining proper concentrations of the resident Golgi proteins and lipids.

### Vesicle formation, tethering, and fusion

The process of forming a vesicle is initiated by the binding of activated small GTPases from the Arf/Sar1 subfamily to the membrane, which recruit coat proteins. As coat proteins polymerize, they form a cage that enhances membrane curvature to begin the formation (or budding) of the vesicle 1, 2, 3.

There are at least three types of coats that function at different locations in and around the Golgi: COPI, COPII, and clathrin 4, 5, 6, 7, 8, 9. Each of these coats, though composed of different subunits, all follow the same basic steps outlined above. COPI‐coated vesicles are mostly utilized in retrograde transport, both within the Golgi and from the Golgi to the ER. The COPI coat is composed of seven different protein subunits αCOP, βCOP, β’COP, γCOP, δCOP, εCOP, and ζCOP 7. The COPII coat is composed of Sec23 and Sec24 on the inside of the coat and Sec13 and Sec31 on the outside and is mostly involved in anterograde ER–Golgi transport 9. Clathrin coat is composed of clathrin heavy and light chains and one of several adaptor complexes and functions in anterograde trafficking from the Golgi, retrograde trafficking from PM, as well as trafficking between endosomes and other compartments 8, 10, 11. The clathrin coat works with several different classes of adaptor proteins to give a specificity for packaging of these vesicles 8, 11, 12.

**Figure 2:**
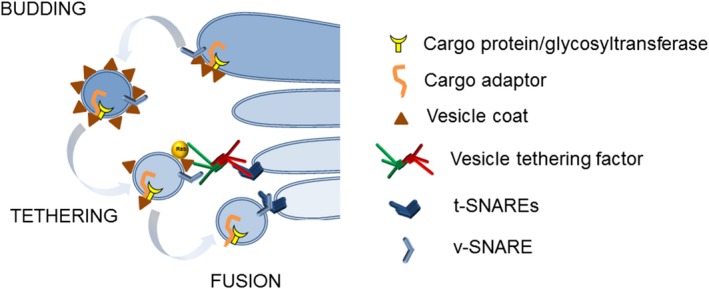
Vesicle formation and fusion events. (a) The coat forms around the budding vesicle and the vesicle eventually buds off the donor compartment. The vesicle is then transported to the acceptor compartment; vesicle is partially uncoated. (b) The Rab protein and remaining coat elements on the vesicle make first contact with the acceptor membrane through tethering proteins; vesicle uncoating is completed. (c) The uncoated vesicle is brought into close proximity to the acceptor membrane where the t‐ and v‐SNAREs form a trans‐SNARE complex to provide the energy needed for membrane fusion to occur.

Shortly after the vesicle buds from the donor membrane it becomes completely or partially uncoated. The coat remnants can then interact with tethering machinery (such as tethering complexes Dsl1 13 and COG 14 before vesicles become fully uncoated 15. Vesicle fusion occurs by interaction between the uncoated vesicle and the target membrane in SNARE‐dependent process (see below). A schematic depicting the major players in vesicle formation, tethering, and fusion is shown in Fig. 2.

Rab GTPases are peripheral membrane proteins that behave as molecular switches—‘turning on or off’ depending on the nucleotide they are associated with. There are ~ 20 different members in the Rab family that associated with the Golgi. Each Rab has a preferred cellular location, with each step of membrane trafficking having different Rabs or Rab combinations. 16, 17, 18, 19. When a Rab binds to GTP it becomes activated, attaches to the membrane, and then recruits other factors (primarily molecular motors and tethers) needed for vesicle fusion.

Prior to fusion vesicles must find their target membrane and be properly aligned. This step is called tethering and is mediated by two different classes of proteins: coiled‐coil tethers (CCTs) 20, 21, 22 and multisubunit tethering complexes (MTCs) 5, 22, 23, 24, 25, 26.

Coiled‐coil tethers, as their name suggests, consist of a long coiled‐coil structure often terminating with a noncoiled‐coil head domain, and, many if not all CCTs function as dimers 20, 22, 24, 27. Most of the known CCTs reside at the Golgi and are often called Golgins 28. Although CCTs all have a similar structure, they vary greatly in size (from ~ 50 to ~ 400 kDa). Due to their elongated structure, CCTs are thought to make first contact with the vesicle, bringing it closer to the target membrane. Supporting this role in trafficking, CCTs interact with SNAREs, Rabs, and other small GTPases located on vesicles and target membranes 23, 24, 25. The binding of tethers to vesicle‐associated Rabs may induce changes in a CCT's structure, generating an ‘entropic collapse force’ that pulls the captured vesicle toward the target membrane 29, 30.

**Figure 3:**
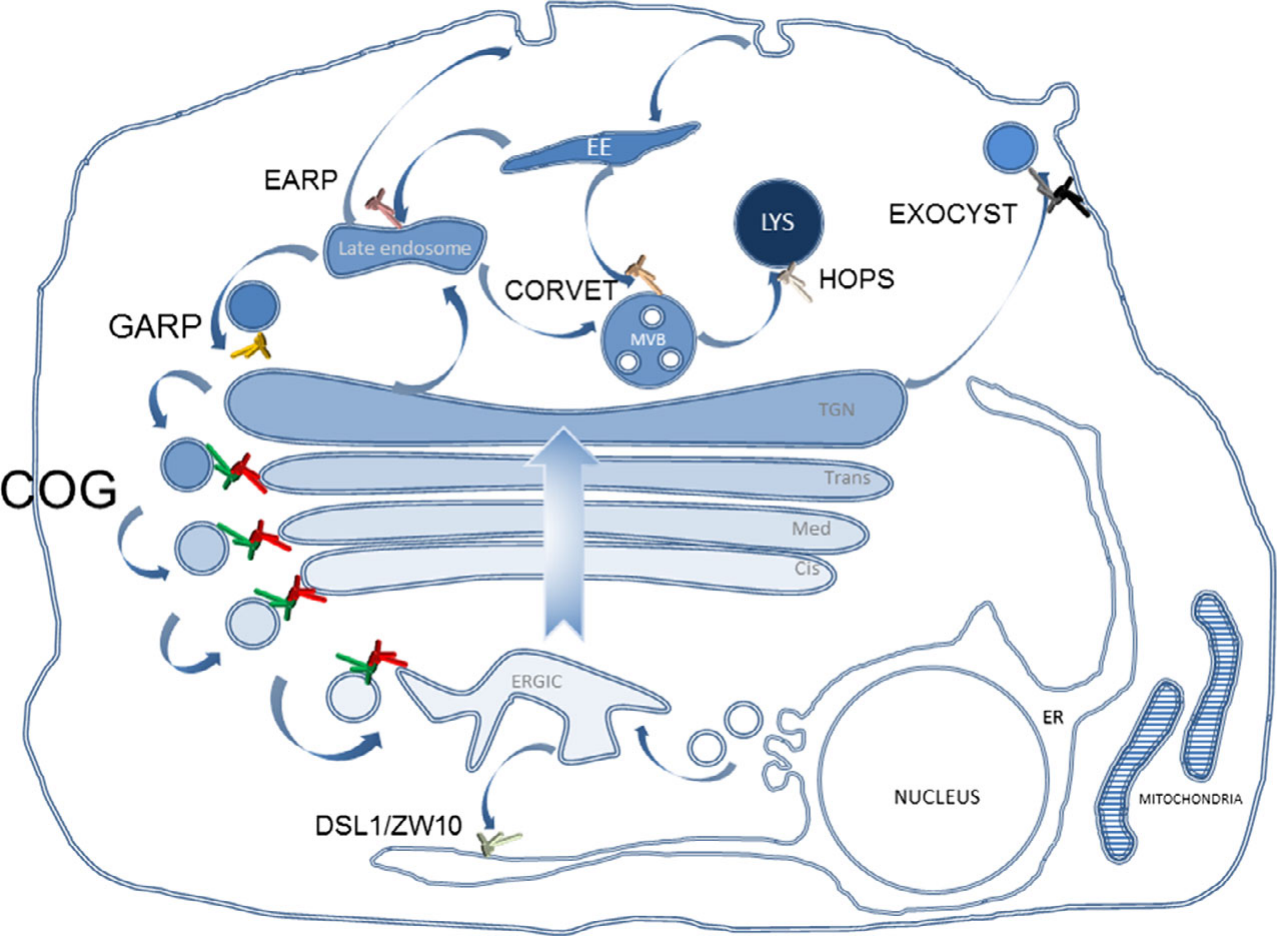
Multisubunit tethering complexes control every step of anterograde and retrograde vesicle delivery in eukaryotic cell.

Multisubunit tethering complexes are generally shorter than CCTs, and are composed of multiple different subunits, which potentially allow them to interact with the fusion machinery in a simultaneous or sequential manner 27, 31. MTCs are subdivided into CATCHR (complexes associated with tethering containing helical rods: Dsl1, COG, GARP, EARP, and exocyst) and non‐CATCHR (TRAPP I, II and III, HOPS and CORVET) complexes based on the structure of their subunits 25. The majority of MTCs interact with Rabs, CCTs, and SNAREs suggesting similar functions for all members within this family. The site of action for different MTCs is depicted in Fig. 3.

SNAREs (soluble N‐ethylmaleimide‐sensitive, factor‐activating protein receptors) are transmembrane molecular machines involved in vesicular fusion 32, 33, 34, 35. SNAREs are localized both on the vesicle and target membrane (v‐ and t‐SNARES). They work in a bundle comprising of four SNARE motifs that are contributed by each of the v‐ and t‐SNARES in the bundle. SNAREs are additionally classified into Qa,b,c‐ and R‐SNAREs based on the amino acid in the 0‐layer, or center, of the SNARE motifs 33, 36, 37. It was proposed that the energy provided by formation of the SNARE complex brings the membranes close together 32, 34, 38, leading to fusion of the vesicle with the target membrane 39, 40.

SM (Sly1/Munc18) proteins assist in vesicle fusion in conjunction with SNAREs. SM proteins can bind to individual Qa‐SNAREs in a closed formation, or assist in zippering of the SNARE bundle by binding to, or ‘clamping’, the trans‐SNARE complex, which likely further facilitates membrane fusion. This regulatory role of SM proteins is believed to give more specificity to the SNARE fusion reaction by promoting correct SNARE pairing while inhibiting incorrect SNARE pairing 35, 41, 42, 43. After the vesicle has merged with the target membrane N‐ethylmaleimide sensitive factor (NSF) and soluble NSF attachment proteins (SNAPs) disassemble the cis‐SNARE complex to recycle the SNAREs for another round of fusion (for review see Refs 44, 45).

## Protein and lipid modifications at the Golgi

While proteins and lipids traverse the Golgi, numerous post‐translational modifications occur including the further processing of N‐glycosylation (which is initiated in the ER), the beginning of mucin‐type O‐glycosylation, and the synthesis of glycolipids 46, 47. Glycosylation employs up to 2% of the proteome, meaning cells expend a large amount of energy ensuring that this crucial process occurs smoothly 48. Glycosylation is dependent upon membrane trafficking not only to bring substrates to the glycosylation machinery for processing but also for the proper localization of glycosylation machinery. Glycosylation results in more diverse protein and lipid structures and aids in folding and function 46.

Glycosphingolipids (GSLs) are the most common type of glycolipids in mammalian cells. Gangliosides, GSLs with sialic acid residues, are enriched in neurons and are important for signaling, cell to cell recognition, and neuronal development and function 49, 50. The Golgi is also an important site for sphingomyelin and phosphatidylinositol 4‐phosphate (PI4P) synthesis 51.

## COG complex function in Golgi trafficking and glycosylation

### COG complex structure and partners

There is a sophisticated membrane trafficking machinery at each cisterna of the Golgi, which helps to facilitate all the processes described above. One particular protein complex that appears to interact with nearly all types of trafficking facilitators throughout the Golgi is the conserved oligomeric Golgi (COG) complex. The COG complex is the major CATCHR vesicle tethering complex at the Golgi. It is a hetero‐octameric complex, with subunits named COG1‐COG8 52 that are subdivided into two subcomplexes (called lobes) named lobe A (COG1‐4) and lobe B (COG5‐8). These lobes exist alone as tetramers in addition to the complete octameric complex 53. COG1 and COG8 form the major bridging interaction between the two subcomplexes and are sometimes viewed as a separate subcomplex 53, 54, 55. The subunits of the COG complex are predicted to form alpha‐helical bundles, which allow for structural flexibility and for dynamic interactions with the trafficking machinery introduced above 56.

Through electron microscopy (EM) studies two main conformations of the COG complex have been uncovered, one after mild fixation with paraformaldehyde and the other unfixed. The unfixed COG complex has an extended and seemingly flexible structure with multiple elongated, curved arms with globular or ‘hook like’ ends. This extended structure is approximately 50–75 nm long 56, 57, 58. The fixed COG complex has a more globular appearance (∼ 37 nm in length) with rod‐like connections between the two main lobes 52. While immuno‐EM experiments revealed that several COG subunits are preferentially localized on the tips of Golgi cisternae 59, 60, recent live cell super‐resolution microscopy studies showed important differences in the localization of COG subcomplexes. Lobe A was found to be preferentially Golgi bound, while lobe B was preferentially localized on vesicles 53.

The COG complex is highly evolutionarily conserved with homologous subunits present in every eukaryotic species 61, 62. COG is most closely related to the exocyst complex, another CATCHR complex that is also composed of eight different subunits 23, 25, 56, 58, 63, 64, 65, 66. Interestingly, COG4 and several exocyst's subunits (EXOC3, EXOC6, and EXOC7) have a homology to the MUN domain of Munc13 67, one of the major priming factors for tethering and fusion of synaptic vesicles 68.

**Figure 4:**
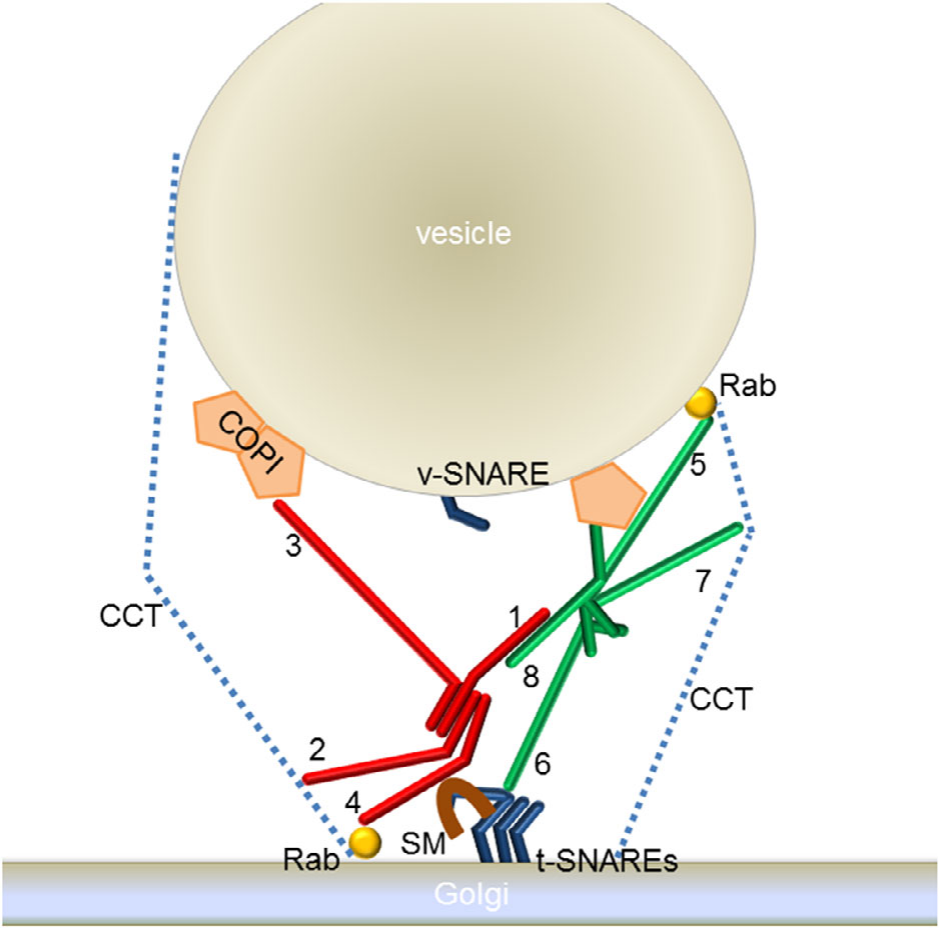
Putative interactions of COG complex with other components of vesicle fusion machinery during vesicle tethering.

The subunits of COG complex form various interaction ‘hubs’ where they specialize in interacting with certain classes of trafficking machinery (i.e., COG4, 5, and 6 interact with several Rab proteins) 69. The COG complex can interact with proteins on the vesicle and the target membrane making it ideal for aligning the two membranes together to allow for SNARE complex formation and vesicle fusion. The COG complex's known interactions are listed in Table 2, though the chronological sequence of these interactions and how they promote vesicular trafficking remain unclear 69. A hypothetical model depicting a functional interaction of the COG complex with a subset of its partners during vesicle tethering is presented in Fig. 4.

Conserved oligomeric Golgi deficient yeast and mammalian cells accumulate Golgi‐derived, ~ 60 nm vesicles called COG complex dependent (CCD) vesicles, presumably due to less efficient tethering 14, 53, 70, 71. Prominently, a massive appearance of CCD vesicles occurs prior to Golgi fragmentation 14, indicating that the accumulation of nontethered Golgi‐derived trafficking intermediates marks the onset of COG complex dysfunction. Moreover, isolated CCD vesicles contain recycling Golgi enzymes and v‐SNARE GS15 and can be tethered *in vitro* in a COG‐dependent reaction 71, 72 confirming COG complex's role as a vesicle‐tethering factor.

### COG‐deficient model systems

The COG complex has been studied in many organisms, from single‐celled *Saccharomyces cerevisiae* to more complex model organisms including *Arabidopsis thaliana*,* Drosophila melanogaster*, and *Caenorhabditis elegans*. Below and in Table 1 we have compiled key findings from all COG deficient organisms described in the literature to compare how COG dysfunction affects different types of eukaryotes both at the cellular and organismal level. These defects are grouped into three categories: altered glycosylation, trafficking abnormalities and protein instability, and morphological aberrations.

**Table 1:** Defects associated with COG complex dysfunctions.

Organism	Mutation	Phenotype	Reference
Yeast, *Saccharomyces cerevisiae*	COG2 (sec35‐1), COG3 (sec34‐2) ts mutants, COG1 Δ, COG5‐8 Δ	Defects in N‐ and O‐glycosylation, mislocalization of Golgi enzymes, growth defects	54, 66, 70, 76, 77, 87, 161, 162, 163
Fungi, *Aspergillus nidulans*	COG2‐ts, COG4‐ts	Abnormal thickness of cell walls, polarization and protein glycosylation. Early Golgi cisternae is not disassembled	164, 165
Plant, *Arabidopsis thaliana*	T‐DNA insertions in COG3 and COG8	Defective pollen tube growth, altered Golgi, incorrect deposition of cell wall components	60, 83
Worm, *Caenorhabditis elegans*	COG1‐8 (*cogc1‐8*) KD	Protein glycosylation defect, abnormal migration	80, 81
Fly, *Drosophila melanogaster*	COG5 (*fws*), COG7‐CDG	Failure of cleavage furrow ingression in dividing spermatocytes and failure of cell elongation in differentiating spermatids and disrupted formation and/or stability of the Golgi‐based spermatid acroblast. Neuromotor defects associated with altered N‐glycome profiles, reduction in bouton numbers	79, 94, 166
Fish, *Danio rerio*	COG8 (*ffr*)	Disrupted Golgi complex ultrastructure, impaired absorption of fluorescent lipids	167
Hamster cells, CHO	COG1 KO (*ldlB*), COG2 KO (*ldlC*)	Defects in N‐, O‐, and lipid‐linked glycosylation, unstable alpha‐dystroglycan, defective GM3 synthesis	74, 85, 86, 168, 169, 170, 171
Monkey cells, Vero	COG3 KD	Glycosylation defect, inhibition of Shiga toxin and SubAB retrograde trafficking	120
Human cells, HeLa	COG3, 4, 5, 6, 7, 8 KDs	Golgi fragmentation, glycosylation defects, accumulation, and consequent mislocalization of vesicles containing GEARS around the Golgi, delayed SubAB trafficking, a subset of destabilized glycosyltransferases, golgins and SNARES	14, 55, 103, 116, 118, 129
Human cells, HEK293T	COG1‐8 KOs	Golgi fragmentation, glycosylation defects, accumulation of enlarged endolysosomal structures, destabilized glycosyltransferases, altered Cathepsin D secretion	89, 130, 131, 133
Human mesenchymal stromal cell	COG4 KD	Protein glycosylation defect, inhibition of the mineralization capacity	172
Humans, COG1‐CDG	COG1 (2659‐2660insC)	Cells: defect in both N‐ and O‐glycosylation, reduced levels and/or altered Golgi localization of MAN2A and B4GalT1 Patients: N‐ and O‐glycosylation defects: reduced sialylation and galactosylation, mislocalization and dramatic decrease in α‐mannosidase II and β‐1,4 galactosyltransferase I levels; generalized hypotonia, small hands and feet, straightened bitemporal space, and antimongoloid eyelids, ventricular hypertrophy with diastolic abnormalities, growth retardation with a rhizomelic short stature, mild psychomotor retardation, microcephaly, liver enlargement	98, 173
Humans, COG2‐CDG	COG2 (a *de novo* frameshift mutation [c.701dup (p.Tyr234*)] and a missense mutation [c.1900T>G (p.Trp634Gly)])	Cells: sialylation deficiencies, reduced expression of COG3 and COG4 Patients: severe acquired microcephaly, psychomotor retardation, seizures, liver dysfunction, hypocupremia, and hypoceruloplasminemia	104
Humans, COG4‐CDG	COG4 (R729W), COG4 (G516R)	Cells: reduction in COG3 (50%), COG2 (40%), COG1 (25%), and COG5 (40%) protein levels, COG complex formation seemed to be unaffected, mild Golgi dysfunction (compared to COG7 or COG8‐CDG), Golgi dilatation and fragmentation Patients: Saul‐Wilson syndrome, a rare form of primordial dwarfism with characteristic facial and radiographic features	102, 115
Humans, COG5‐CDG	COG5 (homozygous intronic substitution (c.1669‐15T>C) leading to exon skipping)	Cells: undersialylation of N‐ and O‐glycans Patients: moderate psychomotor retardation with language delay, truncal ataxia and slight hypotonia	110, 166, 174, 175
Humans, COG6‐CDG	COG6 (G549V)	Cells: reduction in STX6 levels, glycosylation defects including reduced sialyation of O‐glycans; decreased activity of B4GALT1 but normal import of UDP‐galactose into the Golgi, reduced protein levels of COG5 (55%), COG6 (21%), and COG7 (62%), degradation of mRNA encoding COG6, formation of the COG complex affected Patients: microcephaly, chronic inflammatory bowel disease, micronodular liver cirrhosis, severe neurologic disease characterized by vitamin K deficiency, vomiting, intractable focal seizures, intracranial bleedings and fatal outcome in early infancy	176, 177, 178, 179
Humans, COG7‐CDG	COG7 (intronic splice site mutation (c.169+4A>C))	Cells: disruption of multiple N‐ and O‐glycosylation pathways, completely destabilized COG complex Patients: growth retardation, microcephaly, hypotonia, adducted thumbs, feeding problems, failure to thrive, cardiac anomalies, wrinkled skin and episodes of extreme hyperthermia, skeletal anomalies and a mild liver involvement	96, 101, 173, 180
Humans, COG8‐CDG	COG8	Cells: deficient in sialylation of both N‐ and O‐glycans, slower brefeldin A induced disruption of the Golgi matrix, reduction in COG1, COG5, COG6, and COG7 protein levels but not COG2, COG3 and COG4, COG5, COG6, and COG7 were also mislocalized Patients: cerebellar atrophy, Elevated blood creatine phosphokinase, Alternating esotropia, psychomotor retardation, failure to thrive, intolerance to wheat and dairy products, lack of bowel or bladder control, dry skin with keratosis pilaris, mild contractures of the lower extremities	99, 100, 103, 107, 108
Humans, TMED6‐COG8 translocation	TMED6‐Cog8 fusion protein	Renal cell carcinoma	181

#### Misglycosylation

Underglycosylation (or hypoglycosylation) is one of the most widely noted defects associated with COG dysfunction. In fact, the COG complex was first discovered when studying the underlying cause of LDLR underglycosylation in mutant Chinese hamster ovary (CHO) cells (these cells were later found to be lacking COG1 (*cog1/*LDLB cells) and COG2 (*cog2/*LDLC cells). These two mutants showed nearly identical hypoglycosylation patterns to one another (immature N‐, O‐, and lipid‐linked glycosylation and reduced sialic acid residues in all glycan structures) 73, 74.

Underglycosylation is also present in COG deficient *S. cerevisiae, D. melanogaster, and C. elegans*. Yeast COG mutants were identified in several independent screens for novel temperature‐sensitive (ts) mutants with defects in trafficking and glycosylation. At the restrictive temperature, *sec34‐1* (*cog3*), *sec35‐1* (*cog2*) 70, *tfi1/cod3* (*cog1), tfi2/cod2 (cog6),* and *tfi3/cod1 (cog4*) accumulated multiple 60‐nm vesicles and exhibited N‐ and O‐protein glycosylation defects^66^, ^75^, ^76^, ^77^. Additionally, some of these mutants accumulated multiple membrane structures (*sec36* (*cog1*) 76) and secreted vacuolar protease 77, indicating severe trafficking and sorting defects.

In *D. melanogaster cog7* mutants, the cellular level of glycolipid GM1 and sialylated proteins was dramatically reduced 78. N‐glycan mass spectrometry (MS) analysis confirmed hyposialylation of N‐glycans, similar to CHO‐COG mutants and COG‐CDG patients (discussed in the next section). Fly COG mutants also displayed increased high‐mannose, paucimannose, and difucosylated structures—indicative of erroneous and incomplete glycosylation at the Golgi 79.

In *C. elegans,* mutants *cog1* and *cog3* 80 were analyzed for N‐glycosylation defects. N‐glycan MS showed that the *cog1* mutant had no tetrafucose structures and less terminal fucosylation, the equivalent of sialylation in *C. elegans*. Additionally, like in the *Drosophila* mutants, there was an increase in high‐mannose and paucimannose structures 81.

Conserved oligomeric Golgi deficient plants 60, 82, 83 were not specifically characterized for altered glycosylation, but had impaired cell wall function which could be due to COG‐related glycosylation defects affecting cell wall integrity.

#### Protein destabilization and trafficking abnormalities

The misglycosylation observed in COG mutants is likely secondary to membrane trafficking defects, since the fidelity of Golgi glycosylation relies greatly on proper localization and efficient membrane trafficking of glycosylation machinery. Indeed, trafficking abnormalities and protein instability (likely due to abnormal trafficking and/or misglycosylation) are present in all COG deficient organisms.

In CHO cells, several proteins were found to be destabilized upon COG depletion. These proteins were called GEARs and include SNAREs (GS28 and GS15), Golgins (CASP, Giantin, and Golgin‐84), a glycosylation enzyme MAN2A1, and Golgi phosphoprotein GPP130. The sensitivity of these was linked to altered COPI trafficking as COG depletion was found to destabilize COPI and COPI depletion caused similar instability in the GEARs 84. Other proteins sensitive to COG subunit depletion include enzymes (SMS1 and CERT) involved in sphingomyelin synthesis 85, 86 linking COG function to lipid homeostasis.

In yeast, COG mutations result in trafficking defects leading to: altered secretion 70, 77; mislocalization of v‐SNAREs (Snc1 66, Sec22p 77, and Bos1p 77); and defective protein sorting (carboxypeptidase Y and Kar2p) 70. Interestingly, overexpression of several trafficking machinery components including a Rab (Ypt1p), an SM protein (Sly1p^E532K^ 77, 87), SNARES (Ykt6p, Bet1p, and Sec22p), and a CCT (Uso1p 87) partially suppress mutant COG phenotypes. This suggested that, in yeast, the COG complex is primarily needed for the efficiency of intra‐Golgi vesicular trafficking and/or some sort of proofreading step and that mass overproduction of other tethering and fusion components can overcome the COG‐dependency of Golgi trafficking. In agreement to this hypothesis, combining COG mutations with mutations in COPI subunits 76, 77, an αSNAP (*SEC17*) 76, an SM protein (*SLY1*) 76, or a t‐SNARE (*SED5*) 77) resulted in synthetic lethality.

In *D. melanogaster cog* mutants, several Golgi and endosomal proteins including Giotto, ATP7a, Rab1, Rab11, and STX16 are mislocalized 78, 88, 89. Interestingly, *Cog7* and *golph3* (a COPI‐interacting protein that may facilitate packaging of glycosylation enzymes 90, 91) double mutants are synthetic lethal, indicating tight functional connections between COG and recycling COPI machinery.

In *C. elegans* COG mutants, altered trafficking led to mislocalization and degradation of glycosylation enzyme MIG‐23 80. The effects of *cog* mutations were further exacerbated by mutations in other trafficking components (the GARP complex and the SNARE GS28) 92, 93.

In *A. thaliana, cog7* mutation perturbed trafficking resulting in the mislocalization of COPI subunits, ERD2 (KDEL receptor homolog), EMP12 (a COPI cargo protein), GAUT14 (a glycosylation enzyme involved in pectin synthesis), and pectin 60. In addition, *cog7* plants have stunted growth, which could be the result of altered secretion. Similarly, impaired protein secretion was observed in *cog* barley mutants 83.

#### Morphological and growth abnormalities

There are additional defects seen in COG deficient organisms that are more ‘structural’ both at the cellular and organismal level. This section details several observations in COG deficient organisms spanning from abnormal membrane accumulation to altered neuronal function, infertility, and lethality that could not be directly tied to trafficking or glycosylation defects. In yeast, deletions of lobe A subunits COG2, 3, and 4 were lethal, while COG1 KO had a severe growth defect. Lobe B mutants lacked visible growth defects and had fairly normal intracellular morphology in contrast to COG1 KOs and *cog‐2‐ts* mutants which accumulated abnormal internal membranes 66. COG deficient male flies were sterile 78, 94 due to abnormal spermatogenesis resulting from defective cytokinesis 78, 94. Spermatids had a fragmented Golgi and acroblast (a Golgi‐related organelle), and defects in flagellar formation. The life span of *cog7* mutants was reduced compared to wild‐type animals, and neuromotor defects were observed. Additionally, the neuromuscular junctions in these animals were altered, showing a reduced number of boutons 79. Similar neuronal defects were found in *cog1* mutants, indicating that both lobes of the COG complex are needed for the optimal development and function of the neuronal system 89. In worms, COG deficiency affects gonadal formation resulting in reduced proliferation 80. In plants, COG dysfunction causes a number of dysmorphic phenotypes ranging from alterations in the shoot apical meristem and dwarfed *cog7* organisms to male sterility and defects in cell wall components in *cog3* and *cog8* mutants 60, 82. The Golgi in the pollen of these plants was dilated and/or fragmented into mini stacks.

Collectively, and irrespective of the organism, these studies highlight the importance of the COG complex for proper glycosylation, Golgi integrity, proper localization, and stability of selected group of Golgi proteins. Globally, COG also appears to play a role in fertility, neuronal function, and viability.

### Conserved oligomeric Golgi‐congenital disorders of glycosylations

Conserved oligomeric Golgi‐related disorders are also present in humans, though these mutations are relatively rare and give rise to complex pathologies. In humans, COG mutations result in a COG specific, type‐II Congenital Disorders of Glycosylation (or a COG‐CDG for short) 95. Mutations in seven of the eight COG subunits (COG3 being the exception) have been identified as CDG causing 95, 96, 97, 98, 99, 100, 101, 102, 103, 104, 105, 106, 107, 108. CDGs are a very heterogeneous group of disorders, caused by a wide variety of altered gene products, and can result in defects in N‐ glycosylation alone or N‐, O‐, and lipid‐linked glycosylation 106. COG‐CDG patients have misglycosylation of N‐ and O‐linked glycoproteins and glycolipids, which are categorized as CDG‐multiple pathway disorders. The COG complex is different from most proteins whose mutations cause CDGs because COG is primarily a vesicle‐trafficking regulator and not a glycosylation enzyme or sugar transporter, making its impact on glycosylation a secondary effect 95, 109.

The first COG‐CDG patients demonstrated hypotonia, hepatomegaly, microcephaly, loose wrinkled skin, and progressive jaundice that presented soon after birth 96. To date, nearly 70 COG‐CDG patients have been identified 95, 96, 98, 99, 102, 106, 109, 110, 111 (Table 1). COG‐CDG patients share many of the same symptoms, ‘irrespective of the affected subunit’, although some mutations have a milder phenotype than others 106. COG‐CDG patients suffer from severe, multisystemic symptoms that primarily affect the nervous system and liver, perhaps because these organs rely more heavily on secretory traffic and/or glycosylation 112, 113 Other noted defects include: lack of eye muscle control, heart defects, spleen enlargement, skeletal abnormalities, and issues with recurrent infections 106, 109, 112, 114.

Conserved oligomeric Golgi‐CDG mutations are quite heterogeneous in their effect on the disrupted protein, with some patients having no detectable mutant protein, while others have truncations or reductions of the mutant protein. This heterogeneity makes comparisons of different subunit contributions to overall function in human cells difficult.

Interestingly, a new and distinct COG‐related disorder was recently identified involving a heterozygous mutation for COG4. These patients have Saul‐Wilson syndrome (a rare skeletal dysplasia), caused by a *de novo* amino acid mutation in COG4. This mutation does not decrease COG4 protein amount, so it is not a deficiency *per se*, though COG function is distorted. This mutation gives rise to an increase in traffic from the Golgi to the ER and a decrease in ER to Golgi traffic resulting in altered Golgi size and morphology, though glycosylation, surprisingly, remains normal aside from misglycosylation of the proteoglycan decorin 115.

### COG deficiency in human cells

In order to better understand the complex effects of COG loss in humans at the cellular level and to understand the contribution of different subunits to overall COG function, immortalized cell lines have proven useful, as they are readily available and easy to propagate and genetically manipulate. Here, we describe efforts to better understand the role of the COG complex in humans through studies using knockdown (KD), knock‐sideways, and knockout (KO) approaches in HeLa and human embryonic kidney (HEK) cells.

#### HeLa cells

##### Glycosylation

Efforts to better understand how the COG complex affects glycosylation gained impetus as more and more COG‐CDG patients were identified (Table 1) 95, 96, 97, 98, 99, 100, 101, 102, 104, 107, 109, 111. HeLa KDs were used to complement studies in patient fibroblasts. To better characterize COG's role in glycosylation as a whole complex and as the contribution of the two subcomplexes, KDs of COG3, COG5, and COG7 in HeLa cells were created. All KDs resulted in glycosylation defects 55, 71, implicating both lobes of the COG complex in maintaining glycosylation fidelity. A combination of lectin binding and N‐glycan MS analysis was then employed to further study COG malfunction‐induced misglycosylation in four separate COG subunit KDs (two from each lobe). These assays showed defects in *medial* and *trans*‐Golgi enzymes 116, 117, N‐glycan MS showed no major differences in high‐mannose N‐glycans, but did reveal variations in sialylation depending on the depleted subunit (decreased sialylation in COG3 and COG4 KDs; minor increase in COG6 and COG8 KDs). Another study assessing COG3 and COG7 KDs in HeLa cells found terminal sialyation to be affected in both 116, 118.

Glycosylation enzymes MAN2A1, MGAT1, and GalNAcT2 14, 55, 71, 119 were rapidly mislocalized in COG3 KDs, suggesting that mislocalization of the Golgi glycosylation machinery is the main reason for faulty glycosylation in COG deficient cells. Prolonged COG3 KD led to degradation of MAN2A1, indicating that mislocalization to vesicles precedes degradation of COG sensitive proteins 71. MAN2A1 and B4GALT1 stability was reduced in COGKDs. All COG sensitive enzymes were mislocalized to vesicle‐like structures 3 days after KD of either lobe A or lobe B subunits 116.

##### Trafficking and Golgi abnormalities

The mislocalization of enzymes suggests that all COG KDs in HeLa cells likely result in impaired retrograde trafficking that affects retention of Golgi enzymes. This notion was supported by the resistance of the Golgi glycosylation enzymes to be relocalized to the ER upon Brefeldin A and Sar1 DN mediated collapse of the Golgi into the ER (an assay used to indirectly test retrograde Golgi‐ER trafficking efficiency) with the greatest delay being for the *medial*/*trans*‐Golgi‐localized enzymes 116. Retrograde PM–Golgi–ER trafficking of Shiga and SubAB toxins was also dramatically impaired in COG3 KD cells 14, 120.

Several studies to determine a complete set of COG protein partners 119, 120, 121, 122, 123, 124, 125, 126, 127, 128 revealed that the COG complex interacts with all classes of Golgi trafficking proteins, supporting the notion for the central role of the COG complex in regulation of intra‐Golgi retrograde trafficking (for the summary of these interactions see Table 2 and review 69).

**Table 2:** COG partners in mammalian cells.

Partner (interacting region)	COG subunit or assembly (interacting regions)	Evidence for interaction	Reference
Vesicular coat			
β‐COP	COG complex, COG2, COG5, COG8	Co‐IP	14, 128, 135
Rabs			
Rab1a	COG4, COG6	Y2H, *in vitro*	135
Rab1b	COG6	Y2H	135
Rab2a	COG5	Y2H	135
Rab4a	COG4, COG6	Y2H, *in vitro*	135
Rab6a	COG6	Y2H, *in vitro*	135
Rab10	COG6	Y2H	135
Rab14	COG6	Y2H	135
Rab30	COG4 (aa 1–186)	Y2H, co‐IP, *in vitro*	135, 144
Rab39	COG5	Y2H	135
Rab43	COG6	Y2H	135
CCTs			
USO1/P115(HR2)	COG2 (aa 613–669)	Co‐IP, Y2H	53, 121, 124, 128
GOLGA5/Golgin‐84 (aa 340–456)	COG2, COG7	Co‐IP, *in vitro*	124, 135
GOLGA2/GM130	COG complex, COG2, COG3, COG5	Co‐IP, Y2H	121, 124
GOLGB1/Giantin	COG complex,	Co‐IP	121
CUX1/CASP	COG2, COG8	Y2H	135
TMF1 (aa 801–1091)	COG1, COG6	Y2H	135
Trafficking complexes			
RINT1	COG1 (aa 1–93)	Co‐IP	126
BLOC1S1	COG	Co‐IP	182
SNAREs			
STX5	COG complex COG4 (aa 84–153), COG6 (aa 76–150) COG8	Y2H, co‐IP	119, 123, 124, 127, 128, 129
GOSR1/GS28	COG4, COG7	Co‐IP, *in vitro*	14, 124, 140
BETL1/GS15	COG complex	Co‐IP, *in vitro*	53
STX6 (aa 161–234)	COG6 (aa 76–150)	Co‐IP, GST pull‐down, Y2H	125, 127
GOSR2/GS27	COG6 (aa 76–150) COG8	Co‐IP, Y2H	127, 129
SNAP29	COG6 (aa 76–150)	Co‐IP, Y2H	127
VTI1 (aa 121–193)	COG4 (aa 1–231, 232–785) COG8	Co‐IP, *in vitro*	129, 140
STX16 (aa 227–302)	COG4 (aa 1–231), COG7	Co‐IP, *in vitro*	140
SM proteins			
SCFD1/SLY1(aa 1–81)	COG4 (aa 1–84)	Co‐IP, *in vitro*	123
VPS45	COG4 (aa 1–231), COG7	Co‐IP, *in vitro*	140
Others			
ATP7A	COG complex	Co‐IP	89
PI(4,5)P2	COG1, COG4, COG6	Liposome flotation	183

##### Intracomplex interactions

COG subunit KDs in HeLa cells showed that the COG complex's intralobe subunits are codependent on each other for their stability, with the exception of COG8 being tolerant of lobe B subunits loss 55, 71, 127, 128, 129. Upon COG3 or COG4 KD, lobe B subunits are no longer Golgi localized but can still associate with membranes showing that lobe A contributes to but is not solely responsible for membrane localization of the other COG subunits. In COG7 KD cells, COG8 was displaced from the Golgi region, but lobe A stayed on the Golgi membrane, indicating that lobe B is not responsible for lobe A's association with the Golgi, or membrane attachment 128. In knock‐sideways assays, mitochondria relocalized lobe A could recruit newly synthetized lobe B subunits away from the Golgi, but not vice‐versa. This was not true in an inducible knock‐sideways model wherein the complex was already assembled on the Golgi before mitochondrial relocalization was attempted, showing that once in the complex, the COG subunits have a tight association with the Golgi 128.

#### HEK239T knockouts

To better ascertain the contribution of each lobe to overall COG function without dealing with variations in KD efficiencies, COG KOs were created for each subunit using CRISPR/Cas9 approach. Surprisingly, all COG KO cells had similar glycosylation, trafficking, and morphological defects irrespective of the lobe or subunit affected 130, 131. All KO cell lines were uniformly deficient in a subset of *cis/medial/trans*‐Golgi glycosylation enzymes and each had nearly abolished binding of Cholera toxin to the PM, likely as a result of defects in lipid glycosylation. Further characterization of each KO cell line revealed defects in Golgi morphology, retrograde trafficking and sorting, and decreased sialylation and fucosylation, but severities of these defects varied according to the affected subunit. Lobe A and Cog6 subunit KOs displayed a more severely distorted Golgi structure, while COG2, 3, 4, 5, and 7 KOs had the most hypoglycosylated form of Lamp2, a heavily N‐ and O‐glycosylated protein whose shift in electrophoretic mobility is used as a readout for hypoglycosylation. These results led to the conclusion that every subunit is essential for mammalian COG complex function in Golgi trafficking, though to varying extents, perhaps due to different interaction ‘hubs’. COG KO cells also had altered sorting and secretion of Cathepsin D as well as morphological changes to the endosomal/lysosomal system.

**Figure 5:**
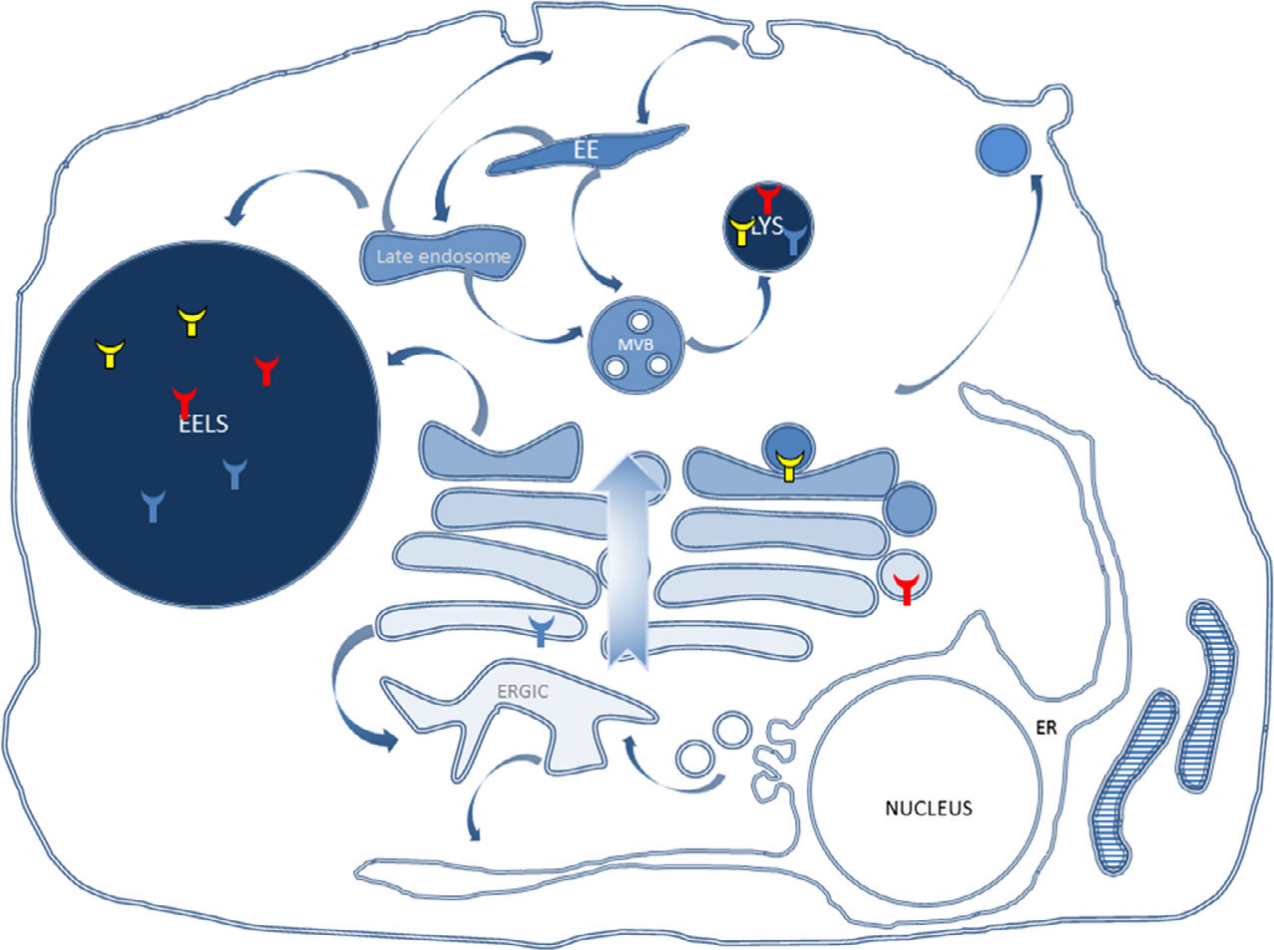
Alterations in secretory/endocytic compartments and intracellular trafficking pathways in a COG depleted cell.

Conserved oligomeric Golgi KOs from each lobe were then compared to other glycosylation mutants [MGAT1 KO, GALE KO, and GALE/MGAT1 double knockouts (DKO)] to decipher which of the COG KO phenotypes were the result of misglycosylation and which were not 132. The KO of MGAT1 and GALE created early and late blocks in N‐glycosylation, respectively. GALE KO also prevented O‐glycosylation by removing available GalNAc. The results were that only a subset of COG KO phenotypes were mimicked by hypoglycosylation alone. Phenotypes not copied by MGAT1 KOs, GALE KOs, or GALE/MGAT1 DKOs include: a severely fragmented Golgi structure, delayed PM–Golgi–ER retrograde trafficking, altered TGN sorting and increased secretion, and accumulation of enlarged endolysosomal structures (EELSs) 133(Fig. 5).

Alterations to the endolysosomal system were further explored to reveal more about the nature of the EELSs. These vacuoles were found to mimic some properties of normal late endosomes/lysosomes such as having an acidic lumen and a mix of endolysosomal membrane proteins (CD63, Lamp2, Vamp7, Rab7, Rab9, and Rab39), but lacking active lysosomal proteases. Lipid homeostasis was perturbed in COG KO cells and some key Golgi lipids, including cholesterol and PI4P, were mislocalized to the EELS's membrane. Furthermore, tested Golgi resident proteins were found to undergo degradation in EELSs. Intriguingly, the maintenance of the EELSs was dependent on GARP activity showing interplay between the two complexes to regulate Golgi and endosomal homeostasis 134.

### Models for COG complex structure and function

A few different models for the COG complex function during vesicle tethering have been proposed 56, 57, 59, 60, 128, 135. In each of these models, the COG complex has a central role in orchestrating membrane trafficking but the stage at which the COG complex is involved differs.

The first model proposed was the docking model that utilizes the entire assembled COG complex. In this model, transport vesicles are initially loosely tethered by long CCTs and then by the COG complex to ensure firm docking. This model is supported by *in vitro* reconstitution experiments 52, 72, 136 in which purified assembled COG complex showed twofold stimulation of vesicle fusion reaction. It is also in agreement with recent models proposed for the HOPS tethering complex 137, 138, but fails to explain the existence of membrane‐attached COG subcomplexes 53, 139 and the dispensability of lobe B subunits in yeast cells.

The second model proposed was the ‘SNARE stabilization’ model. This model was derived from evidence of SNARE protein instability in COG depletion studies 103, 119, 123, 125, 140 (See COG deficiency in human cells), which led to the interpretation that the COG complex's interaction with v‐ and t‐SNAREs may not only contribute to increased SNARE stability but also help SNARE complex assembly. In this model, the COG complex does not directly tether incoming vesicles, but mostly serves to stabilize, catalyze, and possibly proofread the vesicle fusion machinery. The model accounts for the MUN‐like domain in COG4 67 but does not explain extended structural features of the COG complex and multipronged interactions between COG complex subunits with all classes of Golgi trafficking regulators.

**Figure 6:**
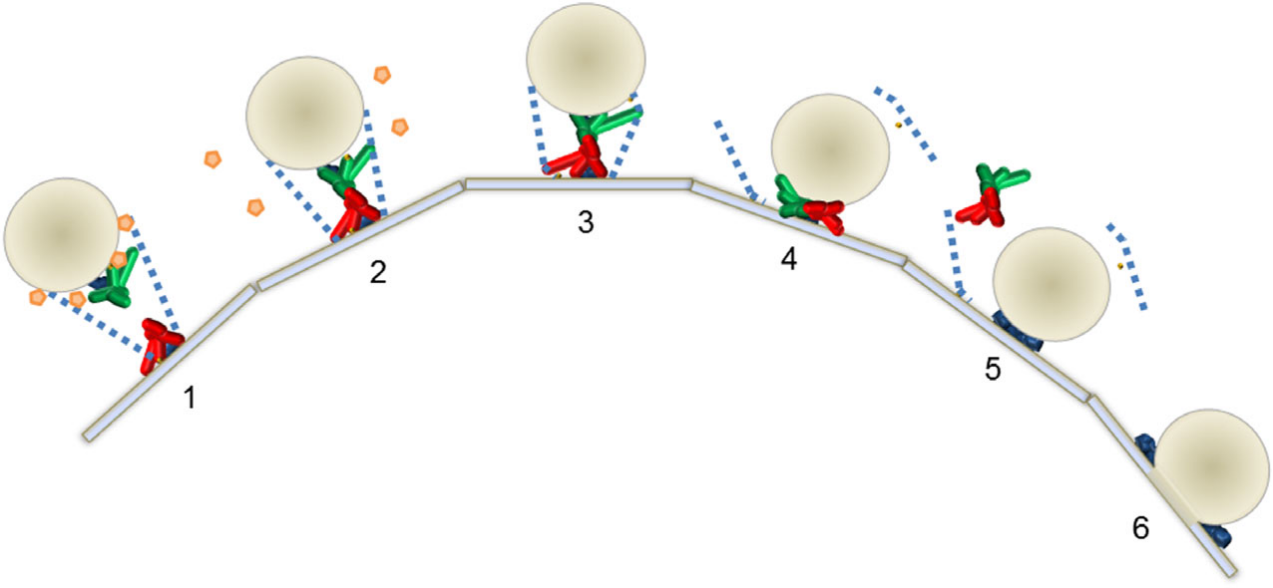
The assembly/disassembly model for COG‐dependent vesicle tethering. (1) COG subcomplexes, lobe A and lobe B, are associated with the Golgi and vesicular membranes, respectively. CCTs mediate initial tethering and bring the vesicle close to the target (Golgi) membrane. COG interacts with the coat that is partially present on the vesicle. (2, 3) The interaction between lobe A and lobe B results in the formation of the entire COG complex and brings the vesicle even closer to the Golgi rim. During this step, the vesicle also gets completely uncoated. (4) COG facilitates alignment of v‐ and t‐ SNAREs leading to the formation of trans‐SNARE complex. (5) The COG complex detaches and the vesicle docking on the target membrane is driven by stable SNARE complex formation. (6) Finally, the vesicle fuses with the Golgi membrane and cargo is delivered.

The third model, the ‘assembly/disassembly’ model, tries to reconcile the first two models and other recent findings (Fig. 6). Willett *et al*. 53 proposed that lobe A and lobe B of the COG complex only transiently work together in vesicle tethering and fusion. In this model, lobe A is initially situated on Golgi membranes and interacts with t‐SNAREs, Golgi Rabs, and CCTs, while lobe B is localized on vesicles and interacts with the v‐SNARE and vesicle Rabs. Lobe A and lobe B contact each other when the vesicle is brought to the Golgi membrane through the long‐distance tethering by CCTs. The COG1‐COG8 interaction forms the octameric COG complex, which in turn activates SM protein to align v‐ and t‐SNAREs, facilitating trans‐SNARE complex formation. The octameric COG complex is then displaced and disassembled, allowing for vesicle fusion to occur. This model predicts that both COG subcomplexes are needed for proper vesicle docking and fusion. Evidence for this model came from observations that COG subcomplexes synthesized in reticulocyte cell lysate *in vitro* 57 or coexpressed in HEK293T cells 141 are stable protein assemblies. Membrane‐bound COG subunits in HeLa cells are found in subcomplexes *in vivo* 53. Moreover, yeast membrane‐associated COG subunits formed a variety of small subcomplexes, whereas cytosolic COG subunits existed as octamers 139. Additionally, isolated COG subcomplexes show lobe‐specific pattern of interaction with different protein partners including β‐COP, p115, and STX5 128. The COG complex assembly/disassembly model is in good agreement with several models proposed for the mammalian exocyst 142, 143.

Notably, in this COG model, other vesicle‐localized CATCHR tethers, for instance, the GARP complex, could functionally substitute the lobe B subcomplex. This potential flexibility would explain the nonessential nature of yeast lobe B and a synthetic lethality observed between mutants of COG and GARP tethering complexes. The assembly/disassembly model would also predict that the octameric soluble COG represents an inert pool of the tether that could be initially activated by some ‘COG disassembly’ activity that would dissociate COG into individual lobes. Alternatively, interaction of soluble COG with preassembled Golgi ‘docking stations’ may be sufficient for COG disassembly. In favor of this prediction, it was shown that the N‐terminal parts of COG subunits play a major role in COG assembly and that the N‐terminal region of COG4 is the major hub for protein‐protein interactions with Golgi‐localized STX5, SLY1, and Rab30 meaning these interactions could compete with one another 119, 123, 125, 135, 144.

## Further questions and perspectives

The experimental efforts and results detailed above have provided an insight into detailed COG mediated trafficking at the Golgi and placed emphasis on the role of the COG complex as the master regulator of retrograde Golgi trafficking and proper organismal function but these studies have also raised a new set of question on the specifics of COG complex function. Here, we pose a few of these questions that will be important to answer in the future.

### What is primarily responsible for COG's association with membranes?

Multiple interactions between COG and its Golgi partners predict the existence of a pool of the COG complex that is permanently attached (‘glued’) to the Golgi periphery *via* a subset of its protein–protein interactions. This COG pool remains primed for a new round of vesicle docking/fusion, activated by a specific ‘trigger’ on approaching vesicles. Supporting this idea, recent fluorescence recovery after photobleaching (FRAP) data indicates that the on/off Golgi kinetic of tested COG subunits is very slow, similar to the FRAP kinetic of transmembrane SNAREs 53 and that both lobe A and lobe B COG subunits remain functional even after their permanent attachment to membranes *via* a Golgi specific transmembrane linker 145. Interestingly, the exocyst when permanently attached to the membrane also remains functional, suggesting that CATCHRs have similar modes of action 146. However, what actually dictates COG's membrane attachment is still unclear.

The COG complex, like a majority of CATCHR complexes, interacts with small GTPases, and it has been proposed that transient interactions with GTP‐loaded Rabs actively recruit COG to the acceptor (Golgi rim) and the donor (recycling intra‐Golgi vesicle) membranes 53, 128, 135. However, depletion of individual Golgi‐localized Rabs has failed to abolish COG localization on the Golgi (VL. unpublished), indicating that no individual Rab is likely responsible for COGs membrane recruitment. Thus, the exact molecular players responsible for COG's membrane recruitment remain unknown. In addition to Rabs, other COG protein membrane partners (SNAREs or other unknown TM proteins) or specific lipids (such as PI4P) could be responsible for COG association with membranes.

### Do COG subunits interact with their partners sequentially or simultaneously and is there a conformational change in the subunits?

COG subunits interact with multiple protein and lipid partners, but the exact nature and sequence of these interactions is still an enigma. One possibility is a sequential mode of interaction between an individual COG subunit and components of tethering/docking/fusion machinery. Supporting this, the same N‐terminal region of COG4 interacts with Rab30, SLY1, and STX5, making simultaneous interaction with all of these partners unlikely. COG could first bind to Rab30 to stabilize vesicle tethering, then switch from Rab30 to SLY1 to activate SM‐SNARE interactions, and finally, bind to STX5 to protect the trans‐SNARE complex from premature SNAP‐NSF‐mediated disassembly.

Another possibility is that at the very first step of a vesicle tethering cycle, COG binds to subunits of COPI coat remaining on the incoming vesicle 77, 135. This COG–COPI interaction may then stimulate COG‐SNARE and COG‐SM interactions, which in turn promote SNARE formation and vesicle fusion. In support to these predictions, another tether, the ER‐localized Dsl1 complex, can bind to COPI, suggesting functional significance for this conserved tether/coat interaction 13, 147, 148, 149. Alternatively, the initial Rab‐COG membrane association could change the conformation of flexible ‘arms’ of COG subunits, allowing them to establish interactions with CCTs, SNAREs, and SM proteins leading to productive vesicle tethering and fusion.

To assist in elucidating the first and following steps of COG‐assisted membrane tethering and fusion, it would be abundantly helpful to reconstitute the COG complex's function *in vitro* using purified components of tethering/fusion machinery.

### How do cells adapt to COG complex malfunction?

The COG complex is evolutionary conserved 62, present in all eukaryotic cells, and lobe A subunits are essential for cell viability in yeast 66. Surprisingly, cultured mammalian cells can tolerate complete KO of any individual COG subunit 130, 133, indicating that higher eukaryotic cells can successfully adapt to COG complex malfunction. What is the mechanism of this adaptation? Does it rely on redundancy of CATCHR tethering complexes or on the flexibility of intracellular trafficking pathways? Does this adaptation involve transcriptional upregulation of specific membrane trafficking components?

### Do heterogeneous/abnormal glycan structures play an additional role in COG KO phenotypes?

Recently, it was questioned whether a severe block in Golgi glycosylation can completely phenocopy the COG KOs 133. Indeed, complete depletion of Golgi enzymes only recapitulated COG KO induced hypoglycosylation, but no other COG KO phenotypes. It is important to note that glycoproteins produced in COG KOs have very heterogeneous glycan structures 130, which could more deleterious to the cell than the complete glycosylation block created in MGAT1/GALE KOs. It is possible that the altered glycan structures found in COG KO cells result in new signaling/structural functions of glycoproteins and/or glycolipids. An example of these abnormal glycans can be seen in the COG KOs unusual affinity for the lectin *Helix pomatia* agglutinin (HPA). HPA binding has been seen in various types of metastatic cancer, and is often correlated with a poorer prognosis, though it is not clear if this is causative or merely correlative 150. Are these altered glycans promoting COG KO cell survival?

### How does COG complex malfunction/depletion affect protein and lipid sorting at the TGN?

The most striking protein and lipid sorting defects in COG KO cells are at the Golgi and post‐Golgi. The *trans*‐Golgi is a major sorting center for the cell and several factors play a crucial role in this process. COG KO‐related *trans*‐Golgi/TGN/endolysosomal malfunction could be a result of changes in ion concentrations (H^+^, Ca^2+^, and Mn^2+^), lipid composition (sphingomyelin, PI4P, cholesterol), mislocalized cargo receptors (SorLA and cab45), or a combination of all three. Further investigation of how these factors are affected in COG KOs could reveal more about the COG complex's role in maintaining Golgi homeostasis.

### How does COG subunit depletion affect endolysosomal homeostasis?

What is the underlying cause of EELS formation in COG KO cells? Is there an altered interplay between COG‐directed intra‐Golgi traffic and lysosomal delivery? The EELS phenotype is rescued upon knocking out either VPS54 or VPS53 subunits of the GARP complex in COG KO cells, which suggests functional cross‐talk between the two MTCs. It is possible that the GARP complex functioning in the absence of COG causes retrograde trafficked Golgi cargo to accumulate at the TGN where it cannot be transported to earlier cisternae of the Golgi resulting in an enlargement of the TGN, which manifests into EELSs 134.

### Are there potential moonlighting roles of COG subunits?

Does the COG complex interact with any other CATCHRs or perform other functions secondary to its primary role in Golgi trafficking? There is some evidence that COG subunits could directly interact with GARP and Exocyst components 60, 82, 83. Additionally, the COG complex shares SNARE partners with the GARP complex 125, 151. Another potential process the COG complex may participate in is autophagy. It has been reported that, in yeast, COG subunits are required for the cytoplasm‐to‐vacuole targeting pathway and for autophagosome formation 152, 153. It will be important to understand the exact role of COG in this process and to investigate if COG plays a role in autophagy in other organisms.

### How and why is the COG complex exploited by pathogens?

Recently the COG has been implicated in allowing for the entrance/survival of multiple intracellular pathogens. *Chlamydia* sp. inclusions recruit both COG and the COG‐interacting SNARE GS15 154. Bacterial growth is reduced in COG KO cells, indicating that hijacking of COG is necessary for continued intracellular survival. The exact mechanism of the COG‐*Chlamydia* relationship is still an enigma. Another intracellular pathogen, *Brucella abortus*, also interacts with the COG complex *via* BspB protein, likely redirecting Golgi‐derived vesicles to Brucella‐containing vacuoles 155. Additionally, the infectivity and/or life cycle of numerous viruses (HIV, Chikungunya Virus, Hepatitis C, Dengue) and toxins (typhoid toxin^156^, SubAB, Cholera toxin, Shiga toxin) somehow depends on COG complex's activity 157, 158, 159, 160. How have these diverse groups of pathogens evolved to rely on COG function? Which functions of COG do they rely on most heavily (i.e., properly glycosylated proteins for binding and entry into the cell, or retrograde trafficking *via* COG to get to their desired location)?

With the wealth of data on the COG complex acquired over the last 35 years we have learned that it is highly conserved and essential for proper glycosylation and membrane trafficking. The COG is responsible for orchestrating a host of partners at the Golgi to harmoniously process, sort and traffic the secretory cargo. Underscoring its significance, mutations in this complex dramatically affect all model species and cells studied. Additionally, in humans, COG mutations result in severe multisystemic CDGs. Yet, we still know little about the specifics of how the COG complex functions, what dictates its localization, or why its depletion affects some organ systems more severely than others. Through technological and scientific advances, we hope these mechanistic questions will be possible to answer in the years ahead.
